# Targeted 'Next-Generation' sequencing in anophthalmia and microphthalmia patients confirms *SOX2*, *OTX2* and *FOXE3* mutations

**DOI:** 10.1186/1471-2350-12-172

**Published:** 2011-12-28

**Authors:** Nelson Lopez Jimenez, Jason Flannick, Mani Yahyavi, Jiang Li, Tanya Bardakjian, Leath Tonkin, Adele Schneider, Elliott H Sherr, Anne M Slavotinek

**Affiliations:** 1 Department of Pediatrics, Division of Genetics, University of California, San Francisco, 533 Parnassus St, Room U585P, San Francisco CA 94143-0748 USA; 2 Broad Institute of Harvard and MIT, Cambridge MA Massachusetts General Hospital, Boston, Massachusetts USA; 3 Department of Neurology, University of California, San Francisco, San Francisco, California 94143-0114 USA; 4 Division of Genetics, Department of Pediatrics, Albert Einstein Medical Center, Philadelphia, Pennsylvania 19141 USA; 5 Vincent J. Coates Genomics Sequencing Laboratory (GSL) QB3/University of California, Berkeley USA B206 Stanley Hall MC 3220 Berkeley, CA 94720-3220

**Keywords:** anophthalmia, microphthalmia, next-generation sequencing, *SOX2*, *OTX2*, *FOXE3*

## Abstract

**Background:**

Anophthalmia/microphthalmia (A/M) is caused by mutations in several different transcription factors, but mutations in each causative gene are relatively rare, emphasizing the need for a testing approach that screens multiple genes simultaneously. We used next-generation sequencing to screen 15 A/M patients for mutations in 9 pathogenic genes to evaluate this technology for screening in A/M.

**Methods:**

We used a pooled sequencing design, together with custom single nucleotide polymorphism (SNP) calling software. We verified predicted sequence alterations using Sanger sequencing.

**Results:**

We verified three mutations - c.542delC in S*OX2*, resulting in p.Pro181Argfs*22, p.Glu105X in *OTX2 *and p.Cys240X in *FOXE3*. We found several novel sequence alterations and SNPs that were likely to be non-pathogenic - p.Glu42Lys in *CRYBA4*, p.Val201Met in *FOXE3 *and p.Asp291Asn in *VSX2*. Our analysis methodology gave one false positive result comprising a mutation in *PAX6 *(c.1268A > T, predicting p.X423LeuextX*15) that was not verified by Sanger sequencing. We also failed to detect one 20 base pair (bp) deletion and one 3 bp duplication in *SOX2*.

**Conclusions:**

Our results demonstrated the power of next-generation sequencing with pooled sample groups for the rapid screening of candidate genes for A/M as we were correctly able to identify disease-causing mutations. However, next-generation sequencing was less useful for small, intragenic deletions and duplications. We did not find mutations in 10/15 patients and conclude that there is a need for further gene discovery in A/M.

## Background

Anophthalmia is found in 1 in 5,000 to 10,000 individuals and is a devastating birth defect because of severe visual impairment [1]. Genetic testing to identify the cause of anophthalmia and/or microphthalmia (A/M) is frequently requested. The transcription factor *SOX2 *is mutated in 10-20% of patients with bilateral A/M and genomic sequencing and deletion analysis of this gene is the first test to determine the cause of severe bilateral A/M [2,3]. However, the remaining pathogenic genes implicated in A/M, such as *OTX2 *or *GDF6*, are each mutated in a small percentage of patients and more than 60% of patients with A/M do not receive a molecular diagnosis after currently available clinical genetic testing (Table 1) [4-16]. In addition, screening of all of the known A/M genes is rarely completed on a clinical basis because there is no currently available panel that covers all of the known genes.

**Table 1:** Known Anophthalmia/Microphthalmia Genes Investigated by Next-Generation Sequencing

Gene	Chromosome location	NCBI Reference Sequence (hg19) mRNA	Exon count	Coding exon count	Estimated Mutation Frequency^4,5^	Inheritance pattern	Reference
*SOX2*	chr3:182912416-182914917	NM_003106.2	1	1	10-20%	Autosomal dominant	6,7
*GDF6*	chr8:97,223,734-97,242,196	NM_001001557.2	3	3	8%	Autosomal dominant	5
*OTX2*	chr14:56337178-56346937	NM_021728.2	5	3	3.3%	Autosomal dominant	8
*VSX2*	chr14:73775928-73799194	NM_182894.2	5	5	2%	Autosomal dominant	9
*FOXE3*	chr1:47654331-47656311	NM_012186.2	1	1	Rare	Autosomal recessive/ Autosomal dominant	10
*CRYBA4*	chr22:25347928-25356636	NM_001886.2	6	5	Rare	Autosomal Dominant	11
*PAX2*	chr10:102495458-102579688	NM_003990.3	11	11	Rare	Autosomal dominant	12
*PAX6*	chr11:31762916-31796085	NM_000280.3	13	10	Rare	Autosomal dominant	13
*SIX3*	chr2:45022541-45025894	NM_005413.3	2	2	Rare	Autosomal dominant	14
*BMP4*	chr14:53486205-53491020	NM_001202.3	4	2	Rare	Autosomal dominant	15

Next-generation sequencing is a recently developed, massively parallel, large-scale sequencing technology that has been used for rapid gene cloning and mutation detection [16]. Next-generation sequencing with exome selection was used to identify the causative genes for Miller syndrome and Kabuki syndrome and has identified mutations in genes for congenital chloride diarrhea and Fowler syndrome [17-20]. Next-generation sequencing has primarily been applied to Mendelian disorders in order to simplify analysis and few birth defects or malformations have been studied. We chose to use this technology to screen known candidate genes in 15 patients with A/M in a pilot study to determine the efficacy of next-generation sequencing for the rapid screening of multiple genes in patient cohorts with birth defects.

## Methods

Written informed consent and genomic DNA samples were obtained from 15 patients with A/M (Table 2) under an approved protocol for the Anophthalmia/Microphthalmia Registry and gene-screening project (Institutional Review Board, Albert Einstein Medical Center). This research adhered to the tenets of the Declaration of Helsinki.

**Table 2:** Summary of Ocular Phenotypes and Next-Generation Sequencing Results in Two Patient Groups

Patient	Right and Left Eye Findings	Additional features	Next generation coding sequence alterations verified by Sanger sequencing in this study
**ANOP1**			
792-505	Anophthalmia	-	-
**792-508**	Anophthalmia	Feeding disorder	**c.313C > T **→ **p.Glu105X in *OTX2***
792-526	Anophthalmia	Learning disabilities	-
792-539	Anophthalmia	Asperger syndrome	-
792-542	Microphthalmia, coloboma, cyst	-	c.24C > G → p.Leu8Leu in *GDF6* c.618C > G (Hz) → p.Ala206Ala in *FOXE3*
792-570	Anophthalmia/Microphthalmia, microcornea, lens opacification	-	c.601 G > A → p.Val201Met in *FOXE3*
792-601	Microphthalmia, chorioretinal colobomas	Hydrocele	-
**09-122**	Anophthalmia	-	**c.542delC **→ p.Pro181Argfs*22 in *SOX2*

**ANOP2**			
792-056A	Microphthalmia	Pervasive developmental disorder	-
**792-518**	Microphthalmia	Hamartoma tuber cinereum	c.124 G > A → p.Glu42Lys in *CRYBA4* **[c.70del20 in *SOX2*]**
792-530	Anophthalmia	Learning disabilities, Arnold-Chiari malformation	-
**792-531**	Anophthalmia	-	c.871 G > A → p.Asp291Asn in *VSX2;* **[c.67-69dupGGC in *SOX2*]**
792-548	Microphthalmia	-	-
**792-563**	Microphthalmia	Arnold-Chiari malformation	**c.720C > A (Hz) **→ **p.Cys240X in *FOXE3***
792-572	Anophthalmia	Learning disabilities, autistic behavior	c.520 G > A (Hz) → p.Ala174Thr in *FOXE3*

Coding sequence variants only are included and mutations are highlighted in bold; (Hz) = Homozygous sequence alteration; the remainder of the sequence alterations were heterozygous.

We divided the 15 patients into two separate groups (Table 2) for sequencing in individual lanes of a flow cell - ANOP1 (8 patients) and ANOP2 (7 patients). The patient samples in each group were pooled without bar-coding. For ANOP1, we sequenced 9 known A/M genes (*FOXE3, SIX3, SOX2, PAX2, PAX6, BMP4, OTX2, VSX2*, and *CRYBA4*; gene order dictated by chromosome location). Our positive control was patient 09-122, who was known to have a *SOX2 *mutation, c.542delC, predicting p.Pro181Argfs*22. For ANOP2, we added *GDF6 *so that 10 known A/M genes (*FOXE3, SIX3, SOX2, GDF6, PAX2, PAX6, BMP4, OTX2, VSX2*, and *CRYBA4*) were sequenced. Our positive control was patient 792-531, with a *SOX2 *sequence alteration, c.67-69dupGGC, resulting in the insertion of an additional glycine residue to the protein.

Primers for amplification of the coding regions were designed with Primer3 http://frodo.wi.mit.edu/primer3/ based on reference sequences from Entrez Gene http://www.ncbi.nlm.nih.gov/gene. The primers used are provided in Additional File 1, Table S1. Polymerase chain reaction (PCR) products for each patient were pooled after estimating concentrations on an ethidium bromide-stained agarose gel and correlating with the size of the amplicon to obtain equal representation of all amplicons in the final mixture. Pooled DNA reactions were column purified (Qiagen, Valencia, CA) and libraries were prepared for sequencing on the Illumina genome analyzer (GA) platform (Illumina, San Diego, CA). We used 32-bp, paired-end reads and the standard sequencing primer (Illumina, San Diego, CA). After library construction, quality was checked using a DNA chip (2100 Bioanalyzer; Agilent Technologies, Santa Clara, CA). The libraries were sequenced at the Vincent J. Coates Genomics Sequencing Laboratory, California Institute for Quantitative Biosciences http://qb3.berkeley.edu/gsl/Home.html. Image analysis and base calling was performed by Illumina pipeline version 1.4 with default parameters (Illumina, San Diego, CA). We first aligned the raw Solexa reads to the reference genome (build 36.3) with MAQ and then rescaled the initial Solexa quality scores to FASTQ format as described in the MAQ's manual http://maq.sourceforge.net/maq-man.shtml[21]. We ran MAQ with its default parameters - albeit with a larger outer distance for a correct read pair (-a 500) and a larger threshold on the sum of mismatching base qualities (-e100). We then used the Genome Analysis Toolkit to further rescale the quality scores of the aligned reads to match empirical error rates as described previously (1000 Genome Project Consortium, 2010) [22]. Briefly, bases were binned by original quality score, machine cycle, and neighboring nucleotide; empirical errors rates were assessed for each bin at non-dbSNP sites; and recalibrated quality scores were assigned to each bin to match the empirical error rate. We then ignored bases with recalibrated quality scores below 20 (Q20) and measured coverage at each targeted base to assess experimental completeness.

We then called variants using Syzygy (J. Flannick, manuscript in preparation), a previously described software package designed to call variants in non-indexed pooled groups of samples [23]. Briefly, the goal of variant calling is to distinguish true variation from sequencing errors. This task is harder in the pooled setting than in the single sample setting because the frequency of a single minor allele in the pool (1/2N, where N is the number of samples in the pool) can approach the raw error rate of Solexa reads (1% at Q20 bases). To call a variant at a genomic position, Syzygy compares the likelihood that a variant exists in the pool to the likelihood that all non-reference sequence bases at the position are due to sequencing errors. At positions where the log of the likelihood ratio is above 3, Syzygy calls a variant and then uses an expectation-maximization (EM) algorithm to determine the number of samples in the pool who carry the variant. Syzygy further declares as high-confidence variants that (1) have statistically indistinguishable numbers of non-reference bases on reads from the forward (+) and reverse (-) strands of the genome; (2) have positive log-likelihood ratios based solely on reads from the forward strand and also based solely on reads from the reverse strand; and (3) do not occur adjacent to another variant. We used these high confidence variants as our final call set, with all coding variants having likelihood ratio > 3.0 on both forward and reverse strands.

Coding sequence variants were verified by Sanger sequencing on genomic DNA or whole-genome-amplified (WGA) DNA (GenomePlex Whole Genome Amplification kit; Sigma, St Louis, MO). Sequence variants found in WGA samples were confirmed in genomic DNA to exclude the small possibility of errors induced by the WGA.

## Results

**Figure 1:**
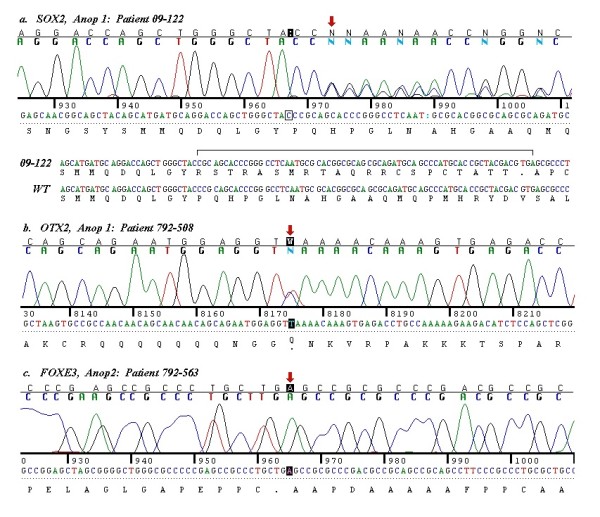
**Mutations in the coding sequence of anophthalmia genes in ANOP1 and ANOP2 patients**. Figure 1A. Chromatogram showing c.542delC in *SOX2 *in patient 09-122, predicted as c.539A > D. Figure 1B. Chromatogram showing c.313C > T in *OTX2*, predicting p.Gln105X in patient 792-508. Figure 1C. Chromatogram showing c. 720C > A in *FOXE3*, predicting p.Cys240X in patient 792-563.

In ANOP1, 13 coding sequence variants were predicted by our method of analysis, 8 of which were listed in the Database of Single Nucleotide Polymorphisms (dbSNP; Table 3). The five predicted sequence variants not in dbSNP were verified by Sanger sequencing as follows: We detected the *SOX2 *mutation, c.542delC, predicting p.Pro181Argfs*22, in patient 09-122, our positive control (Figure 1A). However, this mutation was predicted as a deletion at position c.539 (c.539A > D), 3 bp prior to the location of the actual mutation. c.542delC was not included in a comprehensive recent review of *SOX2 *mutations [7], but the sequence alteration can be presumed to be disease-causing because of likely loss of function, although the deletion occurs after the high mobility group (HMG) domain in the protein. Parental studies were not performed.

**Table 3:** Coding Sequence Variants Predicted by Syzygy and Verified by Sanger Sequencing in the ANOP1 and ANOP2 Libraries

Gene	Position (hg18)	Nucleotide Alteration^a^	Predicted Effect on Protein	dbSNP/1000 Genomes	Sanger Sequencing	Het./ Homo.^b^	Interpretation
***ANOP1***							
*FOXE3*	chr1:47655084	c.510C > T	p.Ala170Ala	rs34082359	Yes; verified	Het.	SNP
*FOXE3*	chr1:47655175	c.601 G > A	p.Val201Met	-	Yes; verified^10^	Het.	SNP
*FOXE3*	chr1:47655192	c.618C > G	p.Ala206Ala	-	Yes; verified^10^	Homo.	SNP
*SIX3*	chr2:45022837	c.90 G > T	p.Ala30Ala	rs78018362	Not done	Het.	SNP
*SIX3*	chr2:45025346	c.942A > G	p.Ala314Ala	rs62840660	Not done	Het.	SNP
***SOX2***	**chr3:182913381**	**c.539A > D^c^**	**Predicted FS^d^**	**-**	**Yes; c.542delC verified**	**Het**.	**Mutation**
*GDF6*	chr8:97242073	c.24C > G	p.Leu8Leu	-	Yes, verified	Het.	Unknown
*PAX2*	chr10:102558862	c.867C > T	p.Asn289Asn	rs1800897	Not done	Het.	SNP
*PAX2*	chr10:102558973	c.978A > C	p.Pro326Pro	rs1800898	Not done	Het.	SNP
*BMP4*	chr14:53487272	c.455T > C	p.Val152Ala	rs17563	Not done	Het.	SNP
***OTX2***	**chr14:56338763**	**c.313C > T**	**p.Glu105X**	**-**	**Yes; verified^8^**	**Het**.	**Mutation**
*VSX2*	chr14:73781636	c.471C > T	p.Ser157Ser	rs35435463	Not done	Het.	SNP
*CRYBA4*	chr22:25351457	c.171C > T	p.Phe57Phe	rs5761637	Not done	Het.	SNP

***ANOP2***							
*FOXE3*	chr1:47655084	c.510C > T	p.Ala170Ala	rs34082359	Yes; verified	Het.	SNP
***FOXE3***	**chr1:47655294**	**c.720C > A**	**p.Cys240X**	**-**	**YES; verified^10^**	**Homo**.	**Mutation**
*SIX3*	chr2:45023323	c.576C > T	p.Arg192Arg	rs182881	Not done	Het.	SNP
*PAX2*	chr10:102558862	c.867C > T	p.Asn289Asn	rs1800897	Not done	Het.	SNP
*PAX2*	chr10:102558973	c.978A > C	p.Pro326Pro	rs1800898	Not done	Het.	SNP
***PAX6***	**chr11:31768060**	**c.1268A > T**	**p.X423Leu** extX*15	**-**	**YES; not verified^24^**	**Het**.	**Mutation**
*BMP4*	chr14:53487272	c.455T > C	p.Val152Ala	rs17563	Not done	Het.	SNP
*VSX2*	chr14:73781636	c.471C > T	p.Ser157Ser	rs35435463	Not done	Het.	SNP
*VSX2*	chr14:73797160	c.871 G > A	p.Asp291Asn	rs75395981	YES; verified	Het.	SNP
*CRYBA4*	chr22:25349282	c.124 G > A	p.Glu42Lys	-	YES; verified	Het.	Unknown
*CRYBA4*	chr22:25351457	c.171T > C	p.Phe57Phe	rs5761637	Not done	Het.	SNP

^a^Nucleotide numbering is according to dbSNP (Database of Single Nucleotide Polymorphisms; http://www.ncbi.nlm.nih.gov/projects/SNP/). Het./Homo.^b ^= heterozygous or homozygous sequence alteration; D^c ^= predicted deletion; FS^d ^= frameshift.

We predicted c.313C > T in *OTX2*, causing p.Gln105X and a premature stop codon just after the homeodomain of the 297 amino acid OTX2 protein (Table 3). This mutation was verified with Sanger sequencing in 792-508 (Figure 1B). This mutation is consistent with the loss of function mutations observed in *OTX2 *in A/M patients [8]. Testing of the healthy parents of this child did not show the same mutation [8].

We verified c.601 G > A, predicting p.Val201Met in *FOXE3*, in patient 792-570 (Additional File 2 Figure S1Ai) and a normal parent (Additional File 2 Figure S1Aii). This SNP has not yet been included in public databases, but has previously been reported in both A/M patients and normal controls and is likely to be a polymorphism [10]. We verified c.618C > G, a synonymous alteration resulting in p.Ala206Ala in *FOXE3*, in patient 792-542, who was homozygous for this SNP (Additional File 2, Figure S1B). The SNP has previously been described in both A/M patients and controls [10]. Finally, we verified c.24C > G, predicting a synonymous alteration, p.Leu8Leu in *GDF6*, in patient 792-542 (Additional File 2, Figure S1C). This novel, synonymous sequence alteration was not predicted to have a significant effect on splicing (Automated Splice Site Analysis; https://splice.uwo.ca). Its significance is therefore uncertain, although it is most likely to be a SNP, as it is present in a low frequency (1/10,755 alleles in the Caucasian and African-American populations) in the Exome Variant Server http://evs.gs.washington.edu/EVS/. We were unable to obtain parental samples.

In ANOP2, 12 coding sequence variants were predicted, of which 9 had been documented in dbSNP or the 1000 genomes database (http://www.1000genomes.org/; Table 3). From the 3 sequence alterations that were not in public databases, we verified c.720C > A, predicting p.Cys240X in *FOXE3 *by Sanger sequencing in patient 792-563 (Figure 1C). The patient was homozygous for this published mutation, consistent with the autosomal recessive inheritance described with *FOXE3 *mutations [10].

We verified c.871 G > A, resulting in p.Asp291Asn, in *VSX2 *in patient 792-531 (Additional File 2, Figure S1D). This sequence variant is a SNP present in the 1000 genomes database and in dbSNP. We also verified c.124 G > A, resulting in p.Glu42Lys in *CRYBA4*, in patient 792-518 (Additional File 1, Figure S1E). Sequencing of 90 control chromosomes was normal for this sequence variant (data not shown) and Polyphen-2 http://genetics.bwh.harvard.edu/pph2/ predicted that the alteration was 'possibly damaging', although the position-specific independent counts (PSIC) score was low at 0.438. Parental samples were unavailable and the significance of this sequence variant is not known, although it is likely to be a SNP as the same patient has a pathogenic deletion in *SOX2*. Finally, we predicted c.1268A > T, resulting in p.X423LeuextX*15, a previously described 'run-through' mutation in *PAX6 *[24]. However, we did not verify this sequence change using a forward primer (Additional file 3, Figure S2) and the polyA tail from the *PAX6 *gene prevented sequencing in the reverse direction. This was the only predicted missense alteration that we were unable to confirm.

In ANOP2, patient 792-531 was known to have a three base pair insertion in *SOX2*, c.67-69dupGGC, predicting the insertion of an additional glycine residue at residue 24 after a stretch of five glycine residues at amino acids 19-23 in the wildtype protein (data not shown). Patient 792-518 was also found to have the common *SOX2 *deletion, c.70del20, resulting in p.Asn24fs*88 and premature protein truncation during the course of this work [9]. Neither of these mutations was predicted when we used high likelihood scores in both the forward and the reverse strands at the same base for mutation prediction (data not shown). This methodology would not have detected larger deletions or insertions, as one strand could still contain normal sequence at the base involved in the mutation, depending on the size of the abnormality. We therefore re-checked all of the predictions for deletions and insertions that had a likelihood ratio > 3.0 in either the forward or the reverse strand for *SOX2 *and found that both an insertion and deletion were predicted at nucleotide c.57 G (data not shown). Although this prediction was not precisely at the position of c.67-69dupGGC or c.70del20, Sanger sequencing of this region in the ANOP2 patients would have lead to the verification of the duplication in patient 792-531 and the deletion in patient 792-518 (data not shown). It should also be noted that we used short reads and that a 20 bp deletion in the read size may result in insufficient homology for a read to be accurately mapped to the reference genome. We conclude that our analysis was highly effective for the detection of missense alterations, but less efficient for deletions and duplications, particularly those of a larger size. Our analysis did not predict any large copy number variants (data not shown).

Coverage for the ANOP1 library was high, with 100% of coding sequence of the 9 genes at > 160X coverage (i.e. > 20X for 8 samples) except for exon 6 of *PAX2*, which was not covered by the library (Additional file 1, Table S2). For ANOP2, there was 100% coverage for the coding exons of 8 genes at > 140X (i.e. > 20X for 7 samples). Exon 6 of *PAX2 *was not covered and *FOXE3 *(78.7% > 140X coverage) and *SIX3 *(96.9% > 140X coverage; Additional file 1, Table S2) had reduced coverage for some regions in the ANOP2 library. It is unclear if the reduced coverage has resulted in false negative results. We did detect homozygosity for c.520 G > A, predicting p.Ala174Thr in *FOXE3*, by Sanger sequencing in patient 792-572 whilst sequencing to verify c.720C > A in *FOXE3 *(Table 2; data not shown). The c.520 G > A sequence alteration was novel and was not predicted with the high threshold parameters used, but was deemed likely to be benign by Polyphen-2, with a PSIC score of 0.025 (data not shown).

## Discussion

We were able to use next-generation sequencing to screen multiple pathogenic genes efficiently in a cohort of 15 A/M patients and were able to identify disease-causing mutations in three patients: c.542delC, predicting p.Pro181Argfs*22 in *SOX2 *(positive control), c.313C > T in *OTX2*, predicting p.Gln105X, and c.720C > A, predicting p.Cys240X, in *FOXE3*. All of these alterations are consistent with loss of function and are considered to be pathogenic. We retrospectively identified a known duplication and deletion in *SOX2 *in two further patients using our analysis method with altered parameters that required a high likelihood score in one strand only (data not shown), but the basepair at which the sequence alteration was predicted was not an exact match to the aberrations found by Sanger sequencing. We did not find mutations in the other 10 patients, although our screening was incomplete in that the *GDF6 *gene was not covered in the ANOP1 library and neither library covered *RAX*. However, our studies emphasize the genetic heterogeneity of A/M, the rarity of mutations in some of the known pathogenic genes and the need for further gene discovery.

We encountered several difficulties with our next-generation sequencing methodology. We chose not to bar-code our samples to simplify library preparation, but our lack of bar-coding to distinguish between different samples increased the number of patients in whom Sanger sequencing was needed. Using bar-coding would have simplified our analysis. We identified relatively few novel coding variants that required Sanger sequencing using Syzygy as our analysis tool, which was a strength of the analysis methodology, and we accepted the prediction of previously reported SNPs as likely to be correct. The error rate of massively parallel sequencing has been estimated to be high, at 0.5% per base call [25]. A high false positive rate is more likely when pooling samples and with low expected frequencies of variant detection, and less likely with increased depth of coverage and paired-end reads [25]. In a recent study, only 74 out of 114 predicted mutations or sequence variants (65%) were confirmed with Sanger sequencing [25]. In our much smaller study, we confirmed 8/9 predicted variants (87.5%).

One of the predicted mutations from our next-generation sequencing, c.1268A > T, resulting in p.X423LeuextX*15 in *PAX6 *is especially noteworthy. This mutation has previously been described in four patients with aniridia, without mention of A/M (*PAX6 *homepage; Leiden Open Variation Database; http://lsdb.hgu.mrc.ac.uk/home.php?select_db=PAX6). The mutation results in an alteration to the normal stop codon, resulting in 'run-through' and a later stop after a further 15 amino acids. It was surprising to us that a mutation for an eye malformation, albeit one distinct from A/M, would be predicted but not verified despite high coverage in that region and the possibility of a low level of mosaicism not detectable by Sanger sequencing cannot be excluded.

## Conclusion

We used next-generation sequencing with a pooled approach to sequence 9 known causative genes in 15 A/M patients. We were successful in identifying three mutations - c.542delC in S*OX2*, resulting in p.Pro181Argfs*22, p.Glu105X in *OTX2 *and p.Cys240X in *FOXE3*. Our analysis methodology resulted in one false positive *PAX6 *mutation that was not verified by Sanger sequencing; we were also unable to detect a small deletion of 20 bp and a duplication of 3 bp, both in *SOX2*. Next-generation sequencing with pooled samples enabled rapid screening of candidate genes for A/M and efficient detection of missense mutations; however, we were less successful in finding small intragenic deletions and duplications.

## Competing interests

The authors declare that they have no competing interests.

## Authors' contributions

NLJ: performed experimental work with PCR and prepared fragments for next-generation sequencing.

JF: performed bioinformatics analysis of results from targeted next-generation sequencing.

MY: performed PCR to verify predicted sequence alterations from Syzygy.

JL: gave advice and experimental help regarding sample preparation for next-generation sequencing.

TB/AS: contributed samples for sequencing.

LT: Sequenced samples with Illumina platform.

EHS: contributed reagents and advice regarding next-generation sequencing.

AMS: experimental design, help with sequencing and analysis of results; wrote the paper.

All authors read and approved the final version of the manuscript.

## Pre-publication history

The pre-publication history for this paper can be accessed here:

http://www.biomedcentral.com/1471-2350/12/172/prepub

## Supplementary Material

Additional file 1 **Table S1**. Amplified Fragments and Primers for Anophthalmia/Microphthalmia Genes. Table S2. Summary of Coverage for Coding Sequence of Anophthalmia/Microphthalmia Genes in ANOP1 and ANOP2 Libraries Click here for file

Additional file 2 **Figure S1**. Single Nucleotide Polymorphisms (SNPs) and Sequence Alterations of Uncertain Significance in Anophthalmia Genes in the ANOP1 and ANOP2 Libraries. Figure S1A. Chromatogram showing c.601 G > A, predicting p.Val201Met in *FOXE3 *in (i) patient, 792-570, and (ii) parent, 792-569. Figure S1B. Chromatogram showing c.618C > G, predicting p.Ala206Ala, in *FOXE3 *in patient 792-542. Figure S1C. Chromatogram showing c.24C > G, predicting p.Leu8Leu, in *GDF6 *in patient 792-542. Figure S1D. Chromatogram showing c.871 G > A, predicting p.Asp291Asn, in *VSX2 *in patient 792-531. Figure S1E. Chromatogram showing c.124 G > A, predicting p.Glu42Lys, in *CRYBA4*, in patient 792-518. Click here for file

Additional file 3 **Figure S2**. Predicted coding sequence variants in ANOP1 and ANOP2 patients that were not verified by Sanger sequencing. Figure S2. Chromatograms showing normal sequence at c.1268A > T, predicting p.X423LeuextX*15 in *PAX6*, in patients from ANOP2. Click here for file
