# Supplementary material for: Targeted 'Next-Generation' sequencing in anophthalmia and microphthalmia patients confirms SOX2, OTX2 and FOXE3 mutations
Source: BMC Med Genet. 2011 Dec 28;12:172. doi: 10.1186/1471-2350-12-172 (PMC3262754; doi:10.1186/1471-2350-12-172)
Supplement: Additional file 1 — Table S1. Amplified Fragments and Primers for Anophthalmia/Microphthalmia Genes. Table S2. Summary of Coverage for Coding Sequence of Anophthalmia/Microphthalmia Genes in ANOP1 and ANOP2 Libraries [file 1471-2350-12-172-S1.DOC]

**Additional file 1, Table S1.**

**Amplified Fragments and Primers for Anophthalmia/Microphthalmia Genes**

| Gene/Fragment | Genomic Location  (hg18) | Size of Genomic Fragment | Size of Amplified Fragment | Primers Used |
| --- | --- | --- | --- | --- |
| ***FOXE3*** | chr1:47654331-47656311 | 1981 bp | 1278 bp | F: ggg gct ggg aga gga aat ta  R: ggg gga cgg aga agg atg ta |
| ***SIX3*** | chr2:45022541-45025894 | 3354 bp | 2939 bp | F: tta cgc cct tcc tcc tct cc  R: ttc ggt ttg ttc tgg gga tg |
| ***SOX2*** | chr3:182912416-182914917 | 2502 bp | 1379 bp | F: gcc aga gga gga ggg aag c  R: ttt tgc gtg agt gtg gat gg |
| ***GDF6 (1)*** | chr8:97,223,734-97,242,196 | 18462 bp | 993 bp | F: tgc gtc ttc ctg cca cac  R: cca cca gcc acc aac aag |
| ***GDF6 (2)*** | chr8:97,223,734-97,242,196 | 18462 bp | 1483 bp | F: gtg gtt acg aag cct ttt gg  R: ggc aag gtg tga aaa tcc at |
| ***PAX2 (1)*** | chr10:102495458-102579688 | 75898 bp | 827 bp | F: agt ctc cgg ccg agt ctt ct  R: ctc caa gat ggg acc tga gc |
| ***PAX2 (2)*** | chr10:102495458-102579688 | 75898 bp | 1599 bp | F: tgg agg tcc acc acc ttt ct  R: tca aga tcc caa ggc tgg at |
| ***PAX2 (3)*** | chr10:102495458-102579688 | 75898 bp | 2249 bp | F: gga gca gat gga tga gga aa  R: cat ccc aca ggg atc gat ag |
| ***PAX2 (4)*** | chr10:102495458-102579688 | 75898 bp | 3267 bp | F: gac cgc tcc atc tct tac ca  R: tca gcc ttt cca ggt tga ag |
| ***PAX2 (6)*** | chr10:102495458-102579688 | 75898 bp | 589 bp | F: agg ggg aca ggc ttg tta gt  R: ggg gtg atg tga agg gtt g |
| ***PAX6 (1)*** | chr11:31762916-31796085 | 33170 bp | 817 bp | F: cag acc aga gca gcc tct tt  R: gtc gcg agt ccc tgt gtc |
| ***PAX6 (2)*** | chr11:31762916-31796085 | 33170 bp | 2450 bp | F: tga gga tgc att gtg gtt gt  R: cag gcc ttc aaa tgc agt ct |
| ***PAX6 (3)*** | chr11:31762916-31796085 | 33170 bp | 1761 bp | F: att tgg gga gtt ggg gtt ac  R: tct caa ggg tgc aga cac ag |
| ***PAX6 (4)*** | chr11:31762916-31796085 | 33170 bp | 1488 bp | F: tag ctc gag gcc caa tct ta  R: aat tcg tgg caa agc ttg tt |
| ***BMP4*** | chr14:53488698-53487356 | 4816 bp | 2572 bp | F: ggg tgg tgt gag gga gaa ga  R: tgg tca aaa cat ttg cac gta a |
| ***OTX2*** | chr14:56341831-56346937 | 5106 bp | 4133 bp | F: aag cct ctg cct cgc cta gt  R: gct ggt ttg tag gcc cct ct |
| ***VSX2 (1)*** | chr14:73775928-73799194 | 23267 bp | 2385 bp | F: ctg cca acc tgc ata gga gt  R: cac tct gtg gct gag ttt gg |
| ***VSX2 (2)*** | chr14:73775928-73799194 | 23267 bp | 1247 bp | F: tga ggg agt ggg aga ttc ag  R: gaa aca gac tgg ggt cag ga |
| ***VSX2 (3)*** | chr14:73775928-73799194 | 23267 bp | 1992 bp | F: gag cct gga gga aca cag ag  R: agg tct gca gga agg aac ag |
| ***CRYBA4 (1)*** | chr22:25348549-25356635 | 8086 bp | 4198 bp | F: tag ccc agt cac tcc tgg ac  R: tgg att cag gct gct caa ct |
| ***CRYBA4 (2)*** | chr22:25348549-25356635 | 8086 bp | 4100 bp | F: tga agg aaa atc ctg cta tgg  R: gac tgt gca cgg acc agt t |

bp = base pairs

**Additional file 1, Table S2.**

**Summary of Coverage for Coding Sequence of Anophthalmia/Microphthalmia Genes in ANOP1 and ANOP2 Libraries**

| **Gene** | **Coverage ANOP1** | **Coverage ANOP2** |
| --- | --- | --- |
| *FOXE3* | chr1: 47654491-47655697  100% >160X | chr1:47,654,491-47,655,590  78.7% >140X |
| *SIX3* | chr2: 45022585-45025523  100% >160X | chr2: 45022654-45025426  96.9% >140X |
| *SOX2* | chr3: 182912669-chr1:182913997  100% >160X | chr3: 182912820-chr1:182913997  100% >140X |
| *GDF6* | - | chr8: 97225743-97242206  100% >140X |
| *PAX2* | chr10: 102495447-10251122  100% >160X  chr10: 102556166-102577533  100% >160X | chr10: 102495447-10251122  100% >140X  chr10: 102556166-102577469  100% >140X |
| *PAX6* | chr11: 31767664-31785059  100% >160X | chr11: 31767664-31785059  100% >140X |
| *BMP4* | chr14: 53486588-53488707  100% >160X | chr14: 53486288-53488707  100% >140X |
| *OTX2* | chr14: 56338029-56342056  100% >160X | chr14: 56338029-56342056  100% >140X |
| *VSX2* | chr14: 73775917-73797896  100% >160X | chr14: 73775917-73797896  100% >140X |
| *CRYBA4* | chr22:25348538-25356591  100% >160X | chr22:25348538-25356591  100% >140X |

All coordinates are given in hg18.
