# Supplementary figures and images for: Targeted 'Next-Generation' sequencing in anophthalmia and microphthalmia patients confirms SOX2, OTX2 and FOXE3 mutations
Source: BMC Med Genet. 2011 Dec 28;12:172. doi: 10.1186/1471-2350-12-172 (PMC3262754; doi:10.1186/1471-2350-12-172)

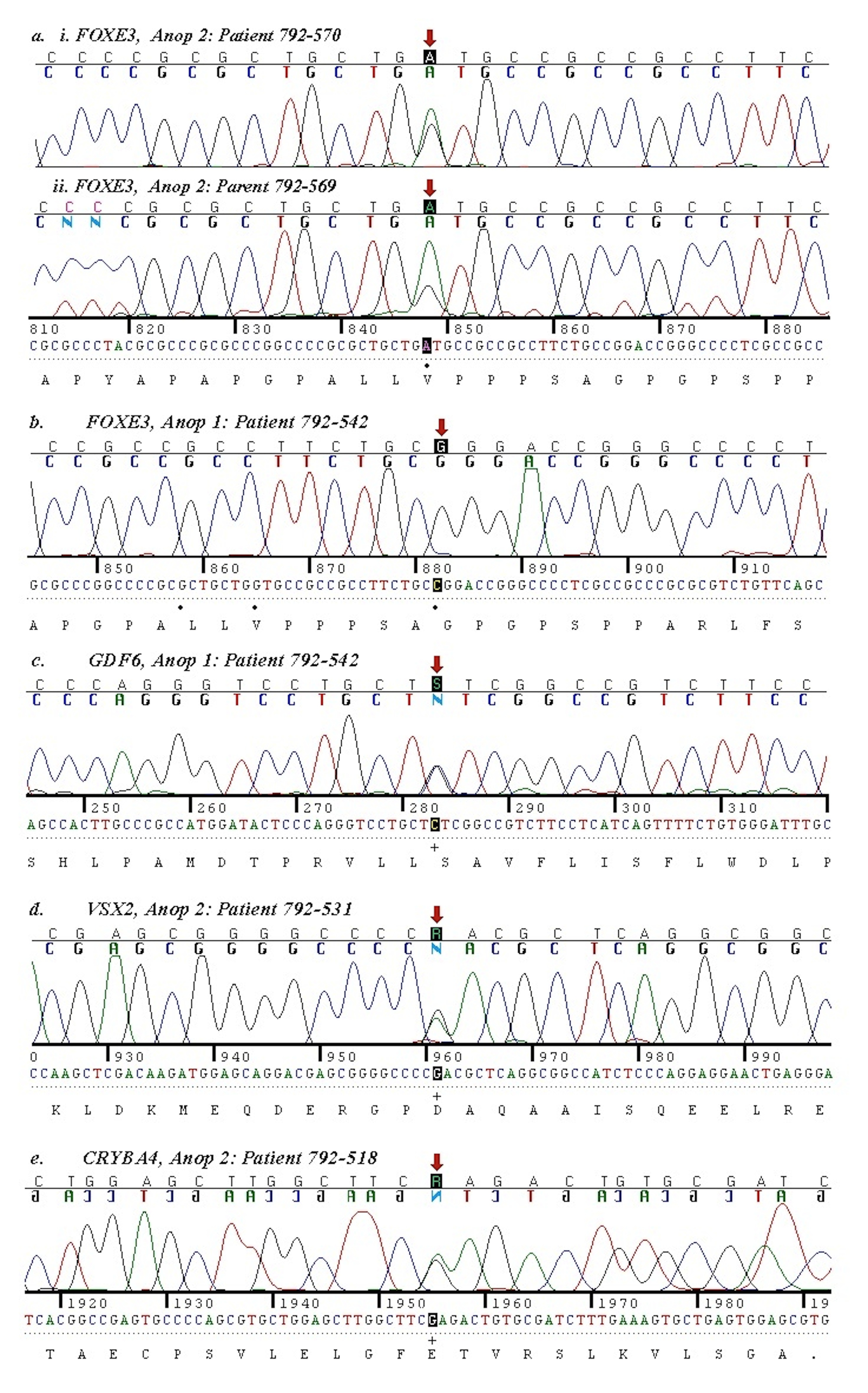

Supplement: Additional file 2 — Figure S1. Single Nucleotide Polymorphisms (SNPs) and Sequence Alterations of Uncertain Significance in Anophthalmia Genes in the ANOP1 and ANOP2 Libraries. Figure S1A. Chromatogram showing c.601 G > A, predicting p.Val201Met in FOXE3 in (i) patient, 792-570, and (ii) parent, 792-569. Figure S1B. Chromatogram showing c.618C > G, predicting p.Ala206Ala, in FOXE3 in patient 792-542. Figure S1C. Chromatogram showing c.24C > G, predicting p.Leu8Leu, in GDF6 in patient 792-542. Figure S1D. Chromatogram showing c.871 G > A, predicting p.Asp291Asn, in VSX2 in patient 792-531. Figure S1E. Chromatogram showing c.124 G > A, predicting p.Glu42Lys, in CRYBA4, in patient 792-518. [file 1471-2350-12-172-S2.JPEG]

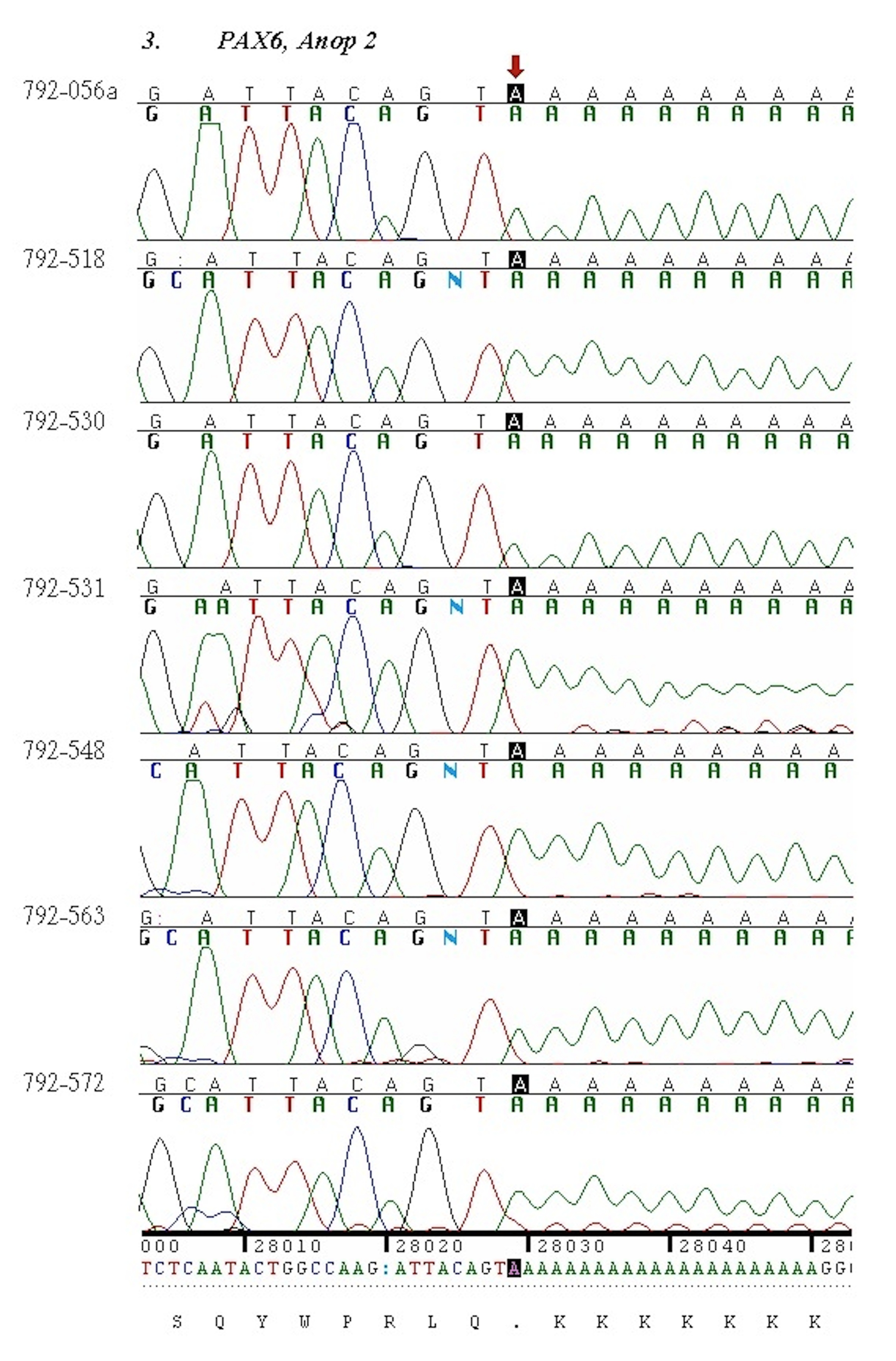

Supplement: Additional file 3 — Figure S2. Predicted coding sequence variants in ANOP1 and ANOP2 patients that were not verified by Sanger sequencing. Figure S2. Chromatograms showing normal sequence at c.1268A > T, predicting p.X423LeuextX*15 in PAX6, in patients from ANOP2. [file 1471-2350-12-172-S3.JPEG]
